# Assessing Validity and Bias of Within-Person Variability in Affect and Personality

**DOI:** 10.1177/01461672231208499

**Published:** 2023-11-22

**Authors:** Farid Anvari, Noëlle Z. Rensing, Elise K. Kalokerinos, Richard E. Lucas, Iris K. Schneider

**Affiliations:** 1 Dresden University of Technology, Germany; 2 University of Cologne, Germany; 3 The University of Melbourne, Parkville, Victoria, Australia; 4 Michigan State University, East Lansing, USA

**Keywords:** emotions, neuroticism, personality, variability, measurement

## Abstract

Within-person variability in affect (e.g., Neuroticism) and personality have been linked to well-being. These are measured either by asking people to report how variable they are or to give multiple reports on the construct and calculating a within-person standard deviation adjusted for confounding by the person-level mean. The two measures are weakly correlated with one another and the links of variability with well-being depend on which measure researchers use. Recent research suggests that people’s repeated ratings may be biased by response styles. In a 7-day study (*N* = 399) with up to five measurements per day, we confirmed that the measures of variability lacked sufficient convergent validity to be used interchangeably. We found only 1 significant correlation (of 10) between variability in repeated ratings of affect or personality and variability in repeated ratings of a theoretically unrelated construct (i.e., features of images). There was very little evidence supporting the response styles hypothesis.

## Introduction

Within-person variability in emotions and personality has been linked to important psychological outcomes such as well-being, depression, self-esteem, and life satisfaction (Block, 1961; Donahue et al., 1993; Fukushima & Hosoe, 2011; Grühn et al., 2013; Houben et al., 2015; Houben & Kuppens, 2020; Kuppens et al., 2007; Nelson et al., 2020). Within-person variability in personality reflects how much people’s personalities, their behaviors, and their emotions fluctuate across contexts and over time (Baird et al., 2017; Campbell et al., 1996; Magee et al., 2018; Marsh, 1993; Rosenberg, 1979). Variability in negative affect in particular is considered an important individual difference given that it is a core component of Neuroticism, a domain of the Big Five personality traits (DeYoung et al., 2007; Eid & Diener, 1999; Eysenck & Eysenck, 1985; John & Srivastava, 1999; Soto & John, 2017).

To assess individual differences in variability, researchers have developed different measures, with the dominant ones being (a) self-reports of perceived variability in the construct and (b) an index of variability (e.g., standard deviation) across repeated self-reports of state levels of the construct. Despite the popularity of these measures, there has also been criticism. Although some measures of within-person variability in affect and personality tend to correlate with well-being, other measures don’t (e.g., Baird et al., 2006). This is concerning because the different measures are supposedly measuring the same thing, and yet they produce different results when examining their links with well-being. Some of the measures of variability also don’t correlate very strongly, or at all, with one another (e.g., Kalokerinos et al., 2020), suggesting that they are either not measuring the same construct or that the measures are contaminated by different sources of measurement error leading to a lack of convergence.

Taken together, conclusions about the association of variability in affect and personality with well-being likely depend on the validity of the measures used. Although it isn’t immediately clear which of the measures is problematic, some research suggests that when items are presented repeatedly, response styles may bias people’s responses to the items (i.e., response style bias). This might generate systematic measurement error for measures of variability that require people to rate their emotions or personality over multiple points in time or across contexts (e.g., Baird et al., 2017). Although the correlations between self-reports of perceived variability and self-reported well-being may be contaminated by common methods bias (Podsakoff et al., 2003), we are focused on the potential for response style bias. As yet, however, the findings in support of response styles bias are inconclusive.

To advance research in both affective science and personality, we examined how strongly several measures of within-person variability in affect and personality correlate with one another, and conducted a confirmatory test of the response styles hypothesis. In the remaining sections of this introduction, we describe the different measures of within-person variability, the evidence for the lack of correlations, or convergent validity, between the different measures, and the problem of response styles. We then introduce our study and hypotheses.

### Measuring Within-Person Variability and the Lack of Convergent Validity

The simplest way to measure within-person variability is to ask people how variable they perceive themselves to be on a given construct—that is, perceived variability. For example, the Affect Lability Scales (Harvey et al., 1989; Oliver & Simons, 2004) and self-report measures of Neuroticism (e.g., DeYoung et al., 2007; John & Srivastava, 1999; Soto & John, 2017) include items that ask people how much they experience variability in their feelings (i.e., affect) either in general or from one moment to the next. Similarly, to measure variability in personality, the Self-Concept Clarity (Campbell et al., 1996), Stability of Self (Marsh, 1993), and Self-Pluralism (McReynolds et al., 2000) scales include questions that ask people to rate how much they believe that their personality varies either across contexts or over time. In the context of the personality trait of self-esteem, the Stability of Self scale has been used as a measure of perceived variability in self-esteem (Webster et al., 2017). Such measures assume that people accurately perceive and report their variability. If people aren’t accurate, then self-reports of perceived variability would have issues with validity.

Another method of measuring variability is to ask people to provide multiple self-reports on a given construct over time and then examine how much people change by calculating the amount of variability on those responses. This is usually done by calculating some variation of a within-person standard deviation. For example, people can report their state levels of negative affect on multiple occasions and researchers can calculate the standard deviation for each person across these multiple occasions (e.g., Eid & Diener, 1999; Houben et al., 2015; Kuppens et al., 2007).

However, the within-person standard deviation of a variable is dependent on the person-level mean of the same variable (e.g., Baird et al., 2006). The person-level mean is derived using people’s responses on the same items that are used to calculate the within-person standard deviation. And so, the maximum possible standard deviation a person can have in their responses depends on their *M*: The closer a person’s mean is to the midpoint of the scale the higher their standard deviation can be; the closer their mean is to the endpoints of a scale the lower the maximum possible standard deviation. That is, people who have a mean closer to the midpoint of a scale are able to show more variability than people with a mean closer to the endpoints. For example, consider a person with a mean of 2 for negative affect on a scale of 1–7. This person is limited in how much variability they can show on the scale by the scale’s floor—they can’t go lower than 1. In contrast, a person with a mean at the midpoint (i.e., 4) can show greater variability because they aren’t limited by the artificial boundaries of the scale’s endpoints. Therefore, the within-person standard deviation is dependent on the person-level mean.

The dependency of the within-person standard deviation on the mean becomes a problem when the distribution of means is positively skewed—that is, most responses are at the lower end of the scale—because the dependency can then result in a spurious correlation between the standard deviation and mean (Baird et al., 2006; Eid & Diener, 1999). For example, when the distribution of means is positively skewed, as is often the case for negative affect (e.g., Dora et al., 2022; Trampe et al., 2015; Zelenski & Larsen, 2000), people with relatively low mean scores can only have smaller standard deviations since most of their ratings will be near the lower end of the scale (reflected in the low mean)—that is, their variability is limited by the artificial endpoint of the scale. In contrast, people with moderate mean scores can have either smaller or larger standard deviations (the same mean score at the midpoint of a scale could be due to either all ratings being at the midpoint or half of the ratings being at the floor and half at the ceiling, and everything in between). With positively skewed distributions, very few people have high scores. This overall pattern can produce a spurious positive correlation between the person-level mean and the within-person standard deviation that is indistinguishable from a correlation that is due to an underlying psychological process. Because the scale endpoints and person-level mean restrict the variability that a person can show, and the means are asymmetrically distributed over the scale, the person-level mean is a potential confound in the correlation of the within-person standard deviation with any other variable. To address this problem, researchers have proposed various methods of adjusting the within-person standard deviation so that the dependence on the mean is removed (e.g., Baird et al., 2006; Mestdagh et al., 2018).

Adjusting the within-person standard deviation has consequences for the relationships typically reported in the literature on within-person variability and well-being. Many psychological outcomes have been linked to variability in affect and/or personality as measured by self-reports of perceived variability or the within-person standard deviation of repeated self-reports given across contexts or over time (Baird et al., 2006; Campbell et al., 1996; Cowan, 2019; Dizén & Berenbaum, 2011; D’Mello & Gruber, 2021; Fukushima & Hosoe, 2011; Grühn et al., 2013; Hanley & Garland, 2017; Houben & Kuppens, 2020; Kamen et al., 2010; Kashdan & Farmer, 2014; Look et al., 2010; Sun et al., 2018; Thompson et al., 2017). But when the within-person standard deviation is adjusted to account for the person-level mean, the correlation of within-person variability with well-being becomes much smaller, regardless of which method of adjustment researchers use (e.g., Baird et al., 2006; Dejonckheere et al., 2019; Fukushima & Hosoe, 2011; Kalokerinos et al., 2020; Magee et al., 2018). Such findings suggest that individual differences in within-person variability in affect and/or personality are less important for well-being than is suggested by findings from research using measures of perceived variability or the unadjusted standard deviation.

These results reflect an important contradiction in research findings. On the one hand, when researchers use self-reports of perceived variability or the within-person standard deviation of repeated reports, variability in affect and personality are relatively strongly related to well-being. On the other hand, when researchers use the adjusted within-person standard deviation, variability in affect and personality are much less strongly related to well-being. This contradiction in research findings is either because self-reports of perceived variability and the adjusted within-person standard deviation in repeated self-reports are capturing different constructs and/or they are contaminated by different sources of measurement error.

If these measures assess the same construct, and they are not differentially impacted by different sources of measurement error, then this could be demonstrated by a strong correlation between the measures (i.e., convergent validity). For negative affect, personality, and even self-esteem, self-reports of perceived variability correlate strongly and positively with the unadjusted within-person standard deviation (Baird et al., 2006, 2017; D’Mello & Gruber, 2021; Eid & Diener, 1999; Grühn et al., 2013; Houben et al., 2015; Sperry & Kwapil, 2020; Webster et al., 2017; Wendt et al., 2020). However, perceived variability in negative affect and personality do not correlate very strongly with the adjusted within-person standard deviation in the same constructs (e.g., Baird et al., 2006, 2017; Hisler et al., 2020; Kalokerinos et al., 2020; Nestler et al., 2021; Sperry & Kwapil, 2020; Wendt et al., 2020). The lack of convergent validity between perceived variability and the adjusted within-person standard deviation is evidence that either the two measures do not capture the same construct or that they have different sources of measurement error which causes their lack of convergence.

From a theoretical perspective, self-reports of perceived variability may reflect self-beliefs whereas variability in repeated self-reports of current experience may reflect actual experience (Conner & Barrett, 2012; Robinson & Clore, 2002). On this account, the two measures of within-person variability capture different constructs—that is, perceived variability versus experienced variability. Alternatively, the two measures may be capturing the same construct but show low convergent validity correlations due to being contaminated by different sources of measurement error. Indeed, recent empirical evidence suggests that repeated self-reports may be biased by response styles.

### The Problem With Response Styles Bias in the Within-Person Standard Deviation

Response styles are tendencies to respond to survey questions in idiosyncratic ways, such as the tendency to use the midpoint or endpoints of a scale or to give a narrow vs. wide range of ratings (Van Vaerenbergh & Thomas, 2013). Recent findings suggest that variability in response styles may bias people’s repeated ratings on the same items, and thus any index of variability derived from people’s repeated self-reports. Baird et al. (2017) had participants give repeated ratings on personality items across contexts (Studies 1 and 2) and daily ratings of personality and positive and negative affect items over 2 weeks (Study 2). Participants also rated their satisfaction with 25 neutral items (e.g., “Your telephone number”) and 4 Simpsons characters on 10 characteristics (Studies 1 and 2), as well as how sunny, windy, rainy, and cold the weather was for each day of the 2-week diary study (Study 2). Baird et al. argued that there were no theoretical reasons for within-person variability as measured by the (adjusted) within-person standard deviation, in contextual and daily personality or daily positive and negative affect, to be related to within-person variability in the ratings of neutral items, Simpsons characters, or the weather. Any correlation between these measures would indicate that the measures are biased by response styles. In both studies, within-person variability in personality and affect were positively correlated with within-person variability in the theoretically unrelated constructs. The findings, except for the weather ratings, were replicated in subsequent studies (Nestler et al., 2021). This suggests that, although response styles affect all self-reports, variability in response styles may bias indices of variability based on people’s *repeated* self-reports only, attenuating the correlation of these measures with self-reports of perceived variability.

However, these findings are inconclusive for two reasons. First, the different measures of response styles did not show strong evidence of convergent validity (i.e., they were not consistently related to each other). Variability in cartoon character ratings was strongly correlated with variability in neutral object ratings in 1 study (*r* = .47; Baird et al., 2017, 1), but this was not consistently found in 2 other studies (*r*s = .02, .15, .18, .19, and .39; Nestler et al., 2021, Studies 1 and 2)—the meta-analytic effect size in Nestler et al. (2021) was statistically significant though not strong (*r* = .28). Similarly, the correlation of variability in weather ratings with variability in neutral objects was small and statistically nonsignificant (*r* = .11; Baird et al., 2017, Study 2). It is therefore unclear whether the different measures are capturing the same construct—that is, response styles—as claimed. It is possible, perhaps, that each of these measures captures a small amount of response styles. It is also possible that the observed correlations in support of response styles bias are unreliable (i.e., false positives) due to the relatively small samples of the studies (*N*s = 93—203)—assuming a true effect size of *r* = .21, the observed correlations in studies would stabilize only with samples of about 238 participants (Schönbrodt & Perugini, 2013).

Second, variability in personality ratings given across contexts and over time may be associated with variability in ratings of neutral objects and cartoon characters for reasons other than response styles. For example, it may be that people whose personalities are more variable (i) tend to vary more in how satisfied they are with different objects and (ii) perceive greater variability in the characteristics of cartoon characters. It was for this reason that Baird et al. (2017) included weather ratings. They argued that any individual differences in weather ratings should reflect response styles, since all participants lived in the same city and completed the diary study during the same period of time. However, the correlations of variability in weather ratings with variability in affect and personality were small (*r*s = .08–.22; Baird et al., 2017). Baird et al. examined the weather during the study period and found that there were floor effects for “rainy” ratings due to very little or no rain on most days, and large fluctuations of temperature that would have strongly impacted variability in “cold” ratings. Baird et al. reasoned that these weather events may have reduced how much the weather ratings were tapping into response styles. But given that this was a post hoc explanation, further confirmatory tests of response styles are warranted. Moreover, it could also be the case that people who experience a lot of within-person variability in affect may also vary in how sunny/cold/rainy they perceive a day to be.

In sum, variability in affect and personality have been linked to important psychological outcomes, including subjective well-being. Variability in negative affect in particular is considered an important individual difference given that it is a core component of Neuroticism, a domain of the Big Five personality traits. There are different methods that researchers use to measure individual differences in within-person variability in affect and personality, including directly asking people to report how variable they perceive themselves to be (i.e., perceived variability) and having people give multiple reports on the construct of interest and calculating a within-person standard deviation based on those multiple reports. Although perceived variability tends to correlate strongly with the within-person standard deviation, this relationship becomes weak once the within-person standard deviation is adjusted to account for confounding by the mean. That is, measures of perceived variability in affect and personality lack convergent validity with the adjusted within-person standard deviation for multiple ratings of the same constructs. One reason proposed to account for the lack of convergence is that people’s repeated ratings for the same items are biased by response styles, which subsequently biases any index of variability calculated using the repeated ratings. However, evidence supporting the response styles hypothesis is inconclusive.

### The Present Research and Hypotheses

We conducted a study to test the convergent validity between perceived variability measures and the adjusted within-person standard deviation for several constructs. Our study included a baseline survey and a 7-day experience sampling phase with up to 5 measures per day. In the baseline survey, we included self-report measures of perceived variability in affect and personality, as well as in self-esteem, and repeated reports of personality across contexts. In the experience sampling phase, we measured positive and negative affect (up to 5 times per day), personality (up to 2 times per day), and self-esteem (once per day) over time. In both the baseline survey and on each day of the experience sampling phase, participants also gave repeated ratings for theoretically unrelated constructs meant to capture response styles—visual features of three sets of images. Using the repeated reports of each construct, we calculated an index of variability in the construct by partially out the mean from the within-person standard deviation, as done in previous work (e.g., Baird et al., 2006, 2017). We had four groups of hypotheses regarding perceived variability and the adjusted within-person standard deviation.

First, if perceived variability in positive affect and negative affect capture the same constructs as an adjusted within-person standard deviation in positive and negative affect, respectively, and the two types of measures are not strongly contaminated by different sources of measurement error, then the measures should be positively correlated (i.e., show convergent validity). Therefore, we tested whether different scales measuring perceived variability in positive and negative affect in the baseline survey were positively correlated with an adjusted within-person standard deviation in positive and negative affect ratings, respectively, in the experience sampling phase.

We hypothesized that there should be a positive correlation between:

Negative Emotionality domain from BFI-2 and the adjusted within-person standard deviation in negative affect ratings (H1a);

Emotional Volatility facet from BFI-2 and the adjusted within-person standard deviation in negative affect ratings (H1b);

Volatility subscale from BFAS and the adjusted within-person standard deviation in negative affect ratings (H1c);

Affect Lability Short Scale and the adjusted within-person standard deviation in negative affect ratings (H1d); Affect Lability Short Scale and the adjusted within-person standard deviation in positive affect ratings (H1e).

Second, if perceived variability in personality and an adjusted within-person standard deviation in personality ratings capture the same construct and aren’t strongly impacted by different sources of measurement error, then they should be positively correlated. Therefore, we tested whether different scales measuring perceived variability in personality in the baseline survey were positively correlated with an adjusted within-person standard deviation in personality ratings given across multiple contexts and over time.

We hypothesized that there should be a positive correlation between:

Self-Concept Clarity Scale and the adjusted within-person standard deviation in contextual personality ratings (H2a);

Self-Concept Clarity Scale and the adjusted within-person standard deviation in daily personality ratings (H2b);

Stability of Self Scale and the adjusted within-person standard deviation in contextual personality ratings (H2c);

Stability of Self Scale and the adjusted within-person standard deviation in daily personality ratings (H2d);

Self-Pluralism Scale and the adjusted within-person standard deviation in contextual personality ratings (H2e);

Self-Pluralism Scale and the adjusted within-person standard deviation in daily personality ratings (H2f).

Third, because one of the self-report measures of perceived variability in personality can also be considered as a measure of perceived variability in self-esteem, we also tested whether perceived variability in self-esteem was positively correlated with an adjusted within-person standard deviation in self-esteem ratings given across multiple points in time (Hypothesis 3).

Finally, we tested whether people’s repeated reports on the same items were biased by response styles. If people’s repeated reports are biased by response styles then there should be a positive correlation between the adjusted within-person standard deviation in the substantive constructs and the adjusted within-person standard deviation in the theoretically unrelated constructs (i.e., the blurriness of images, the color vibrance of images, and white-blackness of grayscale images). Therefore, we tested whether the adjusted within-person standard deviations in personality, positive affect, negative affect, and self-esteem were positively correlated with the adjusted within-person standard deviations in ratings of the images on the baseline survey or ratings of the images on each day of the experience sampling phase.

We hypothesized that there should be a positive correlation between:

The adjusted within-person standard deviation in negative affect ratings and the adjusted within-person standard deviation in image ratings at baseline (H4a);

The adjusted within-person standard deviation in positive affect ratings and the adjusted within-person standard deviation in image ratings at baseline (H4b);

The adjusted within-person standard deviation in contextual personality ratings and the adjusted within-person standard deviation in image ratings at baseline (H4c);

The adjusted within-person standard deviation in daily personality ratings and the adjusted within-person standard deviation in image ratings at baseline (H4d);

The adjusted within-person standard deviation in daily self-esteem ratings and the adjusted within-person standard deviation in image ratings at baseline (H4e);

The adjusted within-person standard deviation in negative affect ratings and the adjusted within-person standard deviation in daily image ratings (H4f);

The adjusted within-person standard deviation positive affect ratings and the adjusted within-person standard deviation in daily image ratings (H4g);

The adjusted within-person standard deviation in contextual personality ratings and the adjusted within-person standard deviation in daily image ratings (H4h);

The adjusted within-person standard deviation in daily personality ratings and the adjusted within-person standard deviation in daily image ratings (H4i);

The adjusted within-person standard deviation in daily self-esteem ratings and the adjusted within-person standard deviation in daily image ratings (H4j).

## Method

### Transparency and Openness

We report how we determined our sample size, all data exclusions, and all manipulations. The data collection procedure was preregistered on the OSF and contains details about all measurements in the baseline survey, the experience sampling study, and an exit survey that was used for a different research question and not reported here (https://osf.io/m9dz5/?view_only=3caeae0e07fd4988b596ecea3d15273a). The design, hypotheses, calculation of the measures, and analyses for this study were also preregistered on the OSF (https://osf.io/jywkt/?view_only=e62e941dc87b43478e7d7c7beff7530a). In this paper, we report only the measures that were used for this study. We state any deviations from, or lack of specifications in, the preregistered protocol. The data and analysis files as well as the Supplemental Materials can be found on the OSF (https://osf.io/rnyd5/?view_only=e15d169cc66e4d60bac7fb9047aec64a).

We analyzed the data using R, version 4.2.3 (R Core Team, 2023) and RStudio Team (2021). We used the packages dplyr version 1.0.7 (Wickham et al., 2021), tidyr version 1.1.4 (Wickham, 2021), matrixStats version 0.61.0 (Bengtsson, 2021), Hmisc version 4.6-0 (Harrell, 2021), corrplot version 0.92 (Wei & Simko, 2021), ltm (Rizopoulos, 2006), lavaan (Rosseel, 2012), multilevelTools, version 0.1.1 (Wiley, 2020), and RVAideMemoire, version 0.9-83 (Herve, 2023). We also used the code for functions relativeSD, maximumVAR, checkInput, and checkOutput from the relativeVariability GitHub repository (Murphy, 2021).

### Participants

We determined the sample size by recruiting the maximum number of participants given resource constraints. We aimed for a total of 400 participants to complete the experience sampling study, which, according to G*Power (Faul et al., 2007), would give the two-sided correlational analyses 95% power to detect *r* = .18. We recruited 1,000 participants from Prolific.co in February 2022 using the representative sampling feature for the United Kingdom (matched on age, sex, and ethnicity), assessed their eligibility (e.g., smartphone with appropriate software update) for the experience sampling study, gave them information about the study, and asked if they were interested in participating. We invited 700 eligible and interested participants to complete the baseline survey. Of the 570 participants who completed the baseline survey, only 1 failed both attention checks and was therefore excluded (in line with the exclusion criteria preregistered in the data collection procedure). The remaining 569 participants in the baseline survey had mean age of 45.53 (15.37) years, ranging from 18 to 84 years; 296 reported gender as female, 269 as male, 2 as other, 1 preferred not to say, and 1 participant had a missing value. We invited participants who had completed the baseline survey to complete the experience sampling study in batches until 400 participants had signed up—these data were collected February–March 2022. One participant from the experience sampling phase had to be excluded from the analyses due to a mixup with the timestamps for the scheduled surveys (e.g., the morning surveys were scheduled after 17:00). The remaining 399 participants had a mean age of 46.02 (14.95) years, ranging from 18 to 84 years; 205 reported gender as female, 192 male, 1 reported as other, and 1 preferred not to say.

### Procedure

We paid participants £4.50 to complete the baseline survey (median of 26 min) consisting of various self-report measures. The baseline survey included two attention check questions. The measures in the baseline survey relevant for this study are detailed under the “Measures” section. At the end of the baseline survey, we provided information about the experience sampling study and asked participants if they still wanted to participate. For the experience sampling surveys, we used the open source SEMA3 app (Koval et al., 2019).

The experience sampling study, which always started on a Tuesday, consisted of five surveys per day and participants received a notification on their mobile phone app to complete each of the surveys. The five surveys were scheduled to be sent to participants, based on their time zone, at random times within 5 time slots between 09:30 and 22:00. The average time between each scheduled survey was 158 min (*SD* = 10 min). More details about the scheduling are in the Supplemental Materials. The relevant measures in each of these surveys are detailed under the “Measures” section. To maximize compliance, we went through a compliance procedure for the experience sampling study for each batch of participants (detailed in the Supplemental Materials) and we paid bonuses based on compliance (structure for bonus payments can be found in the preregistration of the data collection procedure). Participants were paid a total of £11.11 on average, including bonuses. Participants completed an average of 29 and median of 32 surveys in the experience sampling study. During each day of the experience sampling study, we sent participants an invitation on Prolific.co to complete a short survey in which they gave 1 rating for each of 6 images (detailed in the “Measures” section). On the following Tuesday, 1 day after the 7-day experience sampling study had finished for each batch, participants were paid their base payment plus bonuses and sent an invitation via Prolific.co to complete the exit survey.

### Measures

We used (i) measures of perceived variability in several constructs, (ii) repeated reports of the constructs across time and/or contexts to obtain an adjusted within-person standard deviation measure of variability in each construct, and (iii) ratings of three sets of images to obtain an adjusted within-person standard deviation measure of variability in a construct that is theoretically unrelated to any of the other constructs (to capture individual differences in response styles). For all measures, the calculations and reverse scoring of items were such that higher scores reflect greater variability. We also measured self-reported well-being in the baseline survey, though these were not preregistered for the analyses. We calculated the reliability indices for self-reports in the baseline survey using Cronbach’s alpha (α). For the positive and negative affect scales in the experience sampling phase, we calculated both the within- and between-persons reliability using multi-level structural equation modeling (multilevelTools package) which calculates Omega (ω).

#### Variability in Substantive Constructs

Table 1 describes the measures of perceived variability and the within-person adjusted standard deviation in the substantive constructs. Note that the Stability of Self Scale is also a measure of perceived variability in self-esteem (Webster et al., 2017). The positive and negative affect items were partly selected from the Scale of Positive and Negative Experience to tap into the broad domains of positive and negative affect (positive, happy, joyful, negative, sad, angry; Diener et al., 2010) and balanced in activation/arousal with items from other sections of the circumplex structure of core affect (proud, enthusiastic, calm, ashamed, anxious, bored; Yik et al., 2011).

**Table 1.** Measures of Within-Person Variability in the Substantive Constructs.

Measure	Survey	Items	Scale	Reliability
Perceived variability in negative affect				
Negative Emotionality domain (BFI2; Soto & John, 2017)	Baseline	e.g., “Often feels sad”.	1 = disagree strongly, to 5 = agree strongly. Ratings averaged.	α = .92
Emotional Volatility facet (BFI2; Soto & John, 2017)	Baseline	e.g., “Is moody, has up and down mood swings”	As above	α = .85
Volatility subscale (BFAS; DeYoung et al., 2007)	Baseline	e.g., “Get upset easily”.	As above	α = .92
Affect Lability Short Scale (Oliver & Simons, 2004)	Baseline	e.g., “I shift back and forth from feeling perfectly calm to feeling uptight and nervous.”	1 = very undescriptive, to 4 = very descriptive. Ratings summed.	α = .94
Perceived variability in personality				
Self-Concept Clarity scale (Campbell et al., 1996)	Baseline	e.g., “My beliefs about myself seem to change very frequently.”	1 = disagree strongly, to 5 = agree strongly. Ratings averaged.	α = .92
Stability of Self scale (Marsh, 1993)	Baseline	e.g., “I often change from a very good opinion of myself to a very poor opinion of myself.”	As above	α = .83
Self-Pluralism scale (McReynolds et al., 2000)	Baseline	e.g., “People who know me well would say I act quite differently at different times.”	0 = false or 1 = true. Scores summed.	α = .84
Perceived variability in self-esteem				
Stability of Self scale (Marsh, 1993)	See above	See above	See above	See above
Adjusted within-person standard deviation (substantive variables)				
Negative affect	ESM (every survey)	Negative, sad, ashamed, angry, anxious, and bored. e.g., “How **negative** do you feel **right now**?”	1 = not at all, to 5 = extremely.	ω_within_ = .74, ω_between_ = .92
Positive affect	ESM (every survey)	Positive, happy, proud, joyful, enthusiastic, and calm. e.g., “How **positive** do you feel **right now**?”.	As above	ω_within_ = .84, ω_between_ = .95
Contextual personality (BFI-10; Rammstedt & John, 2007)	Baseline	e.g., “is reserved”. Using the BFI-10 items, participants rated how they see themselves (i) among friends, (ii) among family, (iii) when with a romantic partner, and (iv) among strangers.	1 = disagree strongly, to 5 = agree strongly.	n/a
Personality over time (BFI-10; Rammstedt & John, 2007)	ESM (morning and evening surveys)	e.g., “is reserved”.	As above	n/a
Self-esteem (Robins et al., 2001)	ESM (evening surveys)	“Right now I have high self-esteem”.	As above	n/a

BFAS Volatility = Volatility subscale of BFAS; ESM = experience sampling methodology study; BFI2 Volatility = Emotional Volatility facet of BFI2.

We examined the distribution of means for each item used to calculate an index of within-person variability in the constructs (i.e., items for contextual personality, daily personality, daily negative and positive affect, and daily self-esteem). For contextual personality, skewness of items ranged from|0.04| to|1.10| (*M* = 0.48, *SD* = 0.32) and kurtosis from|0.05| to|1.19| (*M* = 0.61, *SD* = 0.32); for daily personality, skewness from|0.07| to|1.07| (*M* = 0.50, *SD* = 0.35) and kurtosis from|0.09| to|1.25| (*M* = 0.84, *SD* = 0.34); for negative affect, skewness from|0.97| to|2.52| (*M* = 1.59, *SD* = 0.72)and kurtosis from|0.61| to|6.80| (*M* = 3.00, *SD* = 2.96); for positive affect, skewness from|0.02| to|0.29| (*M* = 0.13, *SD* = 0.11)and kurtosis from|0.05| to|0.58| (*M* = 0.31, *SD* = 0.20); and for self-esteem, skewness was −0.34, kurtosis −0.67.

To calculate the adjusted within-person standard deviations, we preregistered using the same method as the original studies testing the response styles hypothesis (Baird et al., 2006, 2017). We calculated the mean and standard deviation of each item of the scale for each participant. We regressed the item’s standard deviation onto its mean and square of its mean and retained the residual from the model. We included the square of the mean because the dependency of the standard deviation and mean is curvilinear (Baird et al., 2017). The average of the residuals across all of the items of a scale for a participant provides an adjusted within-person standard deviation of that scale, with higher scores reflecting greater within-person variability. The correlation between the adjusted and the unadjusted within-person standard deviation for each construct was extremely high: contextual personality, *r*_(566)_ = .94; daily personality, *r*_(388)_ = .99; negative affect, *r*_(393)_ = .77; positive affect, *r*_(393)_ = .996; and self-esteem, *r*_(377)_ = .98.

There are also other methods of adjusting for the dependence on the mean. For example, in the Supplemental Materials, we report the results of our tests when using an alternative method of adjustment, namely the relative standard deviation (Mestdagh et al., 2018). Other approaches include mixed effects location scale modeling (Mader et al., 2023) and, rather than adjusting for the mean, correcting for it by adjusting by the mode (Ringwald & Wright, 2022).

#### Response Styles Bias

As a measure of response styles bias, we used the adjusted within-person standard deviations for ratings given on three sets of 14 images each at baseline, and three sets of 2 images rated each day of the experience sampling study. These would provide a measure of within-person variability in a construct that should be theoretically unrelated to any of the other constructs that we measured. Participants rated 14 grayscales from 1 = very white to 7-very black, 14 images varying in blurriness from 1 = very blurry to 7 = very clear, and another 14 images varying in color saturation on how vibrant the colors are, from 1 = not at all to 7 = extremely.

In the baseline survey, the three sets of images were presented in random order so that some participants rated the grayscales first, others rated the blurry images first, and yet others rated the color images first. Within each image set, the 14 images were also presented to participants in random order. We calculated an adjusted within-person standard deviation for ratings on each image set separately using the same method as for the other measures. Therefore, each participant had an index of variability for the grayscales at baseline, the blurry images at baseline, and the color images at baseline. These were weakly but statistically significantly and positively correlated (*r*s = .09, .14, and .25). We combined the three adjusted within-person standard deviations for the images rated at baseline into a single composite index of variability if they were positively correlated so that we had 1 index of variability in the baseline image ratings.

Over the course of the 7-day experience sampling phase, participants rated the images again. On each day, we sent participants a study invitation in Prolific.co and had them rate 6 images, 2 from the grayscales, 2 from the blurry images, and 2 from the color images. At the end of the 7 days, each image was sent only once to each participant. We calculated an adjusted within-person standard deviation for each image set using the same method as for the other measures so that each participant had an index of variability for the daily grayscales, the daily blurry images, and daily color images. These were weakly-moderately and statistically significantly correlated (*r*s = .18, .31, and .32). As with the baseline image ratings, we preregistered combining these into a composite index of variability so that we had 1 within-person standard deviation in daily image ratings.

Given the weak to moderate correlations between the variability scores for the images, we tested the robustness of the results (i.e., for Hypothesis 4) using latent variable models. We loaded the adjusted within-person standard deviation for each set of images at baseline onto a latent factor and we tested Hypothesis 4 with this latent factor instead of the composite score. We did the same for the images rated daily. The results and inferences remained largely the same. We report these results in the Supplemental Materials.^1^

#### Well-Being

The baseline survey contained several self-report measures of well-being that we report for exploratory purposes (i.e., not preregistered). We measured *trait positive* (α = .89) and *negative affect* (α = .93) by asking participants how often during the past month they had felt each of 24 emotions while awake, from 1 = never to 7 = always (Diener et al., 1995). We also included the *Satisfaction With Life Scale* (α = .91) rated from 1 = disagree strongly to 7 = agree strongly (Diener et al., 1985), the *Rosenberg Self Esteem Scale* (α = .93), rated from 1 = disagree strongly to 5 = agree strongly (Rosenberg, 1965), and the *Ruminative Responses Scale* (α = .95), rated from 1 = almost never to 4 = almost always (Treynor et al., 2003).

### Exclusions

We preregistered several methods for dealing with careless responding (Ward & Meade, 2023). For the experience sampling data, we excluded any item (*n* = 422) on which participants responded faster than 650ms and any surveys (*n* = 7) on which participants responded faster than 650ms on more than 50% of the items. Such response times (i.e., well below 1 second) suggest that people were unlikely to be introspecting and reporting their feelings accurately. The mean response time across all items in the experience sampling data was 3630ms (*SD* = 14159), with a median of 2412ms. For the baseline survey, we excluded any participants who failed both the attention checks (*n* = 1) and any participants who gave the same response to all of the items either for trait affect, BFI2, BFI-10, BFAS Volatility subscale, contextual personality items, or any of the 3 sets of images (*n* = 0). Two exclusions were not preregistered. We excluded one participant from all analyses due to the timestamps on the surveys of the experience sampling phase being incorrect (e.g., morning surveys sent in the afternoon). We excluded another participant due to being an extreme outlier (6.33 standard deviations below the mean on the corrected standard deviation measure of within-person variability in negative affect), as noted by an reviewer.

## Results

We had four groups of preregistered hypotheses (H1–4). For each hypothesis, we preregistered using two-sided, pairwise, Pearson’s correlation coefficient tests against a null hypothesis of zero. We did not preregister an alpha cut-off level nor any plan to correct for multiple comparisons. In keeping with convention, we will use the alpha cut-off of .05 (i.e., a 5% type-1 error rate). To adjust the alpha-level for multiple comparisons, and thus maintain the 5% error-rate for each hypothesis test, we will use Bonferroni corrections for each group of hypotheses separately. The Bonferroni method is the strictest method of adjusting for multiple comparisons, insofar as it increases the type 2 error rate (i.e., false negatives or not finding a significant result when there is a true relationship), but this strictness offsets our lack of a priori method for adjustment. A summary of the results of the hypothesis tests is presented in a table at the end of the results section.

### Preregistered Hypothesis 1: Convergence of Measures for Variability in Affect

The first hypothesis was that if perceived variability in positive affect and negative affect capture the same construct as an adjusted within-person standard deviation of positive and negative affect ratings given over multiple time points, respectively, and these are not strongly impacted by different sources of measurement error, then they should be positively correlated (i.e., show convergent validity). Therefore, the adjusted within-person standard deviation in negative affect should be positively correlated with the Negative Emotionality (i.e., Neuroticism) domain from the BFI2 (H1a), the Emotional Volatility facet from BFI2 (H1b), the Volatility subscale from BFAS (H1c), and the Affect Lability Short Scale (H1d), and the adjusted within-person standard deviation in positive affect should be positively correlated with the Affect Lability Short Scale (H1e). Table 2 presents the correlations for these variables. The Bonferroni corrected alpha for Hypothesis 1 is (.05/5) .01.

**Table 2.** Correlations [and 95% Confidence Intervals] Testing Hypothesis 1.

Variable	1.	2.	3.	4.	5.	6.
1. NA variability	*N* = 395					
2. Emotionality	.21 [.11, .30]***	*N* = 568				
3. BFI2 Volatility	.22 [.12, .31]***	.87 [.85, .89]***	*N* = 568			
4. BFAS Volatility	.23 [.14, .32]***	.83 [.81, .86]***	.90 [.88, .91]***	*N* = 568		
5. ALS	.30 [.20, .38]***	.66 [.61, .71]***	.61 [.56, .66]***	.61 [.58, .68]***	*N* = 568	
6. PA variability	.70 [.65, .75]***	.16 [.07, .26]**	.19 [.09, .28]***	.19 [.10, .29]***	.30 [.21, .39]***	*N* = 395

*Note*. Results testing H1a-H1d are in rows 2–5 of the first column, respectively, and H1e is in the 6th row of column 5. NA variability = adjusted within-person standard deviation in negative affect ratings; Emotionality = Negative Emotionality domain of BFI2; BFI2 Volatility = Emotional Volatility facet of BFI2; BFAS Volatility = Volatility subscale of BFAS; ASL = Affect Lability Short Scale; PA variability = adjusted within-person standard deviation in positive affect ratings.

** *p* ≤ .01. ***p ≤ .001.

H1a–H1e were all supported with small to moderate correlations (*r*s ranged from .19 to .30; see Table 2, rows 2–5 of column 1 and row 6 of column 5). Therefore, for both negative and positive affect, there was a small degree of convergent validity between self-reported perceived variability and the adjusted within-person standard deviation. As can be seen in Table 2 (and Table 3), the within-method correlations are stronger than the between-method correlations: self-reports of perceived variability correlate with each other more strongly than with the adjusted within-person standard deviation; the adjusted within-person standard deviation for the different constructs correlate with each other more strongly than with self-reports of perceived variability.

**Table 3.** Correlations [and 95% Confidence Intervals] Testing Hypothesis 2.

Variable	1.	2.	3.	4.	5.
1. Context Pers.	*N* = 568				
2. Daily Pers.	.44 [.36, .52]***	*N* = 390			
3. Self-Concept	.17 [.09, .25]***	.22 [.12, .31]***	*N* = 568		
4. Stability of Self	.15 [.07, .23]***	.18 [.08, .27]***	.89 [.87, .90]***	*N* = 568	
5. Self-Pluralism	.20 [.11, .27]***	.15 [.06, .25]**	.70 [.65, .73]***	.65 [.60, .69]***	*N* = 568

*Note*. Rows 3–5 of columns 1 and 2 show the results for H2a–H2f. Context Pers. = Adjusted within-person standard deviation in contextual personality ratings in baseline survey; Daily Pers. = adjusted within-person standard deviation in personality ratings in the experience sampling (morning and evening) surveys; Self-Concept = ratings on Self-Concept Clarity Scale; Stability of Self = ratings on Stability of Self Scale; Self-Pluralism = ratings on Self-Pluralism Scale.

**p ≤ .01. ***p ≤ .001.

### Preregistered Hypothesis 2: Convergence of Measures for Variability in Personality

The second hypothesis was that if perceived variability in personality captures the same construct as an adjusted within-person standard deviation of personality ratings given over multiple contexts or time points, and the two measures are not strongly impacted by different sources of measurement error, then they should be positively correlated. Therefore, the adjusted within-person standard deviation in contextual personality ratings and the adjusted within-person standard deviation in daily personality ratings should each be positively correlated with the Self-Concept Clarity Scale (H2a and H2b), the Stability of Self Scale (H2c and H2d), and the Self-Pluralism Scale (H2e and H2f). Table 3 presents the results of these tests. The Bonferroni corrected alpha for Hypothesis 2 is (.05/6) .0083.

H2a-H2f were supported but with small to moderate correlations (*r*s ranged from .15 to .21; see Table 3, rows 3–5 of columns 1 and 2). Therefore, for variability in personality, there was some evidence of convergent validity between perceived variability and the adjusted within-person standard deviation.

### Preregistered Hypothesis 3: Convergence of Measures for Variability in Self-Esteem

We also hypothesized that, if perceived variability in self-esteem has convergent validity with variability in self-esteem across time, there would be a positive correlation between the Stability of Self Scale and the adjusted within-person standard deviation in self-esteem ratings in the experience sampling study. The results supported the hypothesis (*r* = .14, 95% CI = [.04, .24], *p* = .005, *N* = 379). Though, once again, the correlation was quite weak.

### Preregistered Hypothesis 4: Response Styles

The final hypothesis was that if people’s repeated self-reports are biased by response styles then variability in the substantive constructs should be positively correlated with variability in the theoretically unrelated constructs (i.e., the image ratings). The adjusted within-person standard deviations in ratings for the images at baseline and ratings for the images given daily should, respectively, be positively correlated with the adjusted within-person standard deviations in negative affect (H4a and H4f), positive affect (H4b and H4g), contextual personality (H4c and H4h), daily personality (H4d and H4i), and self-esteem (H4e and H4j). We preregistered additional sub-hypotheses—that is, that there would be positive correlations between the adjusted within-person standard deviations across all substantive measures, H4k-H4r—but these measures would also be expected to be associated on theoretical grounds. For example, the shared variance of ratings across all personality items, perhaps reflecting a self-evaluative bias (Schimmack, 2019), tends to correlate relatively strongly with ratings for self-esteem and positive and negative affect (e.g., in Musek, 2007, absolute *r*s ranged from .51 to .66). Therefore, the critical tests for systematic measurement error are provided by H4a–H4j. The Bonferroni corrected alpha for the critical tests in Hypothesis 4 (H4a–H4j) is (.05/10) .005. Table 4 presents the results of these tests.

**Table 4.** Correlations [and 95% Confidence Intervals] Testing Hypothesis 4.

Variable	1.	2.	3.	4.	5.	6.	7.
1. Base Image	*N* = 568						
2. Daily Image	.40 [.32, .48] *p* < .001	*N* = 393					
3. NA variability	.10 [−.001, .19] *p* = .053	.13 [.03, .23] *p* = .009	*N* = 395				
4. PA variability	.13 [.03, .22] *p* = .009	.10 [<−.01, .20] *p* = .050	.70 [.65, .75] *p* < .001	*N* = 395			
5. Context Pers.	**.13 [.05, .21]** *p* **= .002**	.07 [−.03, .17] *p* = .160	.40 [.32, .48] *p* < .001	.44 [.36, .51] *p* < .001	*N* = 568		
6. Daily Pers.	.04 [−.06, .14] *p* = .468	−.05 [−.15, .05] *p* = .347	.55 [.48, .61] *p* < .001	.67 [.61, 0.72] *p* < .001	.44 [.36, .52] *p* < .001	*N* = 390	
7. Self-Esteem	.01 [−.09, .11] *p* = .910	−.07 [−.17, .03] *p* = .195	.34 [.25, .43] *p* < .001	.43 [.35, .51] *p* < .001	.21 [.11, .30] *p* < .001	.37 [.28, .46] *p* < .001	*N* = 379

*Note*. Rows 3–7 of columns 1 and 2 show the results for H4a–H4j. Base Image = Adjusted within-person standard deviation in image ratings at baseline; Daily Image = Adjusted within-person standard deviation in daily image ratings; NA variability = Adjusted within-person standard deviation in negative affect; PA variability = Adjusted within-person standard deviation in positive affect; Context Pers. = Adjusted within-person standard deviation in contextual personality ratings in baseline survey; Daily Pers. = Adjusted within-person standard deviation in personality ratings in the experience sampling (morning and evening) surveys; Self-Esteem = Adjusted within-person standard deviation in self-esteem ratings in the experience sampling (evening) surveys.

Out of the critical tests, only H4c was supported (bolded in Table 4). The positive correlation between the adjusted within-person standard deviations in contextual personality and in the baseline image ratings was statistically significant at the corrected alpha level (H4c). Therefore, only 1 of the 10 critical tests supported the hypothesis that people’s repeated self-reports on the same items are biased by response styles (Hypothesis 4).

### Exploratory (Non-Preregistered) Analyses

#### Relationship of Variability Measures With Self-Reported Well-Being

Table 5 reports the correlations of the self-reported well-being measures from the baseline survey with the perceived variability measures (top panel) and the adjusted within-person standard deviations (middle panel). The point estimates and corresponding 95% CIs clearly show that the well-being measures are almost always more strongly correlated with the self-report measures of variability than with the adjusted within-person standard deviation measures of variability.

**Table 5:** Correlations [and 95% Confidence Intervals] of Well-Being with Variability Measures.

Perceived variability (*N* = 568)	SWLS	RSES	Trait NA	Trait PA	RRS
BFI2 Emotionality	−.47 [−.53, −.40]***	−.74 [−.78, −.70]***	.79 [.76, .82]***	−.38 [−.45, −.31]***	.69 [.65, .73]***
BFI2 Volatility	−.27 [−.34, −.19]***	−.51 [−.57, −.45]***	.66 [.61, .71]***	−.16 [−.24, −.08]***	.57 [.51, .62]***
BFAS Volatility	−.25 [−.32, −.17]***	−.49 [−.55, −.42]***	.66 [.61, .71]***	−.18 [−.25, −.09]***	.54 [.48, .60]***
ALS	−.28 [−.36, −.21]***	−.53 [−.58, −.46]***	.63 [.58, .68]***	−.15 [−.23, −.07]***	.70 [.65, .74]***
Self-Concept Clarity	−.38 [−.45, −.30]***	−.66 [−.70, −.61]***	.59 [.54, .65]***	−.29 [−.36, −.21]***	.67 [.62, .71]***
Stability of Self	−.29 [−.36, −.21]***	−.57 [−.62, −.51]***	.52 [.46, .58]***	−.21 [−.28, −.13]***	.58 [.52, .63]***
Self-Pluralism	−.26 [−.34, −.19]***	−.46 [−.52, −.39]***	.49 [.43, .55]***	−.21 [−.28, −.13]***	.54 [.48, .60]***
Adjusted Standard Deviation	SWLS	RSES	Trait NA	Trait PA	RRS
NA variability (*N* = 395)	.01 [−.09, .11]	−.09 [−.18, .01]	.24 [.14, .33]***	.05 [−.05, .15]	.28 [.18, .37]***
PA variability (*N* = 395)	.08 [−.01, .18]	−.05 [−.15, .05]	.21 [.17, .30]***	.13 [.04, .23]**	.26 [.16, .35]***
Context Pers. (*N* = 568)	.01 [−.07, .09]	−.08 [−.16, <.01]*	.16 [.08, .24]***	.09 [.01, .17]*	.24 [.16, .31]***
Daily Pers. (*N* = 390)	.02 [−.08, .12]	−.07 [−.16, .03]	.15 [.05, .25]**	.05 [−.05, .15]	.30 [.21, .39]***
Self-Esteem (*N* = 379)	−.004 [−.10, .10]	−.01 [−.11, .09]	.04 [−.06, .14]	.04 [−.07, .14]	.15 [.05, .25]**
Adjusted standard deviation (partial)	SWLS	RSES	Trait NA	Trait PA	RRS
NA variability (*N* = 395)	.01 [−.09, .11]	−.09 [−.19, .01]	.25 [.16, .34]***	.04 [−.06, .14]	.28 [.19, .37]***
PA variability (*N* = 395)	.08 [−.02, .18]	−.05 [−.15, .05]	.23 [.13, .32]***	.14 [.04, .23]**	.26 [.16, .35]***
Context Pers. (*N* = 568)	−.02 [−.12, .08]	−.08 [−.18, .02]	.19 [.10, .29]***	.05 [−.05, .15]	.23 [.13, .32]***
Daily Pers. (*N* = 390)	.02 [−.08, .12]	−.07 [−.17, .03]	.16 [.06, .25]**	.05 [−.06, .14]	.31 [.21, .39]***
Self-Esteem (*N* = 379)	−.01 [−.11, .10]	.001 [−.10, .10]	.04 [−.06, .14]	.04 [−.06, .14]	.16 [.06, .25]**

*Note*. Correlations between well-being measures (columns) and variability measures (rows). Top panel = perceived variability. Middle panel = within-person adjusted standard deviation. Bottom panel = within-person adjusted standard deviation partial correlations (controlling for within-person adjusted standard deviation in baseline image ratings and daily image ratings). SWLS = satisfaction with life scale; RSES = Rosenberg self-esteem scale; Trait NA = trait negative affect; Trait PA = trait positive affect; RRS = ruminative responses scale; BFI2 Emotionality = Negative Emotionality domain of BFI2; BFI2 Volatility = Emotional Volatility facet of BFI2; BFAS Volatility = Volatility subscale of BFAS; ASL = Affect Lability Short Scale; NA variability = adjusted within-person standard deviation in negative affect ratings; PA variability = adjusted within-person standard deviation in positive affect ratings; Context Pers. = adjusted within-person standard deviation in contextual personality ratings; Daily Pers. = adjusted within-person standard deviation in personality ratings in the experience sampling surveys; Self-Esteem = adjusted within-person standard deviation in self-esteem ratings in the experience sampling surveys.

* *p* ≤ .05. **p ≤ .01. ***p ≤ .001.

#### Partial

Correlations of Adjusted Within-Person Variability Measures With Self-Reported Well-Being

To examine how much the bias of response styles from Hypothesis 4 impacts the variability measures, we tested the partial correlations between the adjusted within-person standard deviation for each construct and its corresponding self-report measure(s) of perceived variability, after removing the variance associated with response styles. For each pair of correlations in these analyses, we controlled for the within-person standard deviation in ratings for both the baseline images and the daily images. These are reported in the next paragraph. Similarly, the bottom panel of Table 5 reports the partial correlations between the self-reported well-being measures from the baseline survey and the adjusted within-person standard deviations, controlling for the adjusted within-person standard deviation in the baseline and image ratings (bottom panel).

From the partial correlation analyses, we found that the adjusted within-person standard deviation in negative affect was significantly positively correlated with (i) the emotionality domain of the BFI2, *r* = .22, 95% CI = [.12, .31], *p* < .001; (ii) the emotional volatility facet of the BFI2, *r* = .23, 95% CI = [.13, .32], *p* < .001; (ii) the volatility subscale of the BFAS, *r* = .23, 95% CI = [.13, .32], *p* < .001; and (iv) the ALS, *r* = .30, 95% CI = [.21, .39], *p* < .001. The adjusted within-person standard deviation in positive affect was significantly positively correlated with the ALS, *r* = .31, 95% CI = [.21, .40], *p* < .001. The adjusted within-person standard deviation in contextual personality was positive correlated with (i) the self-concept clarity scale, *r* = .17, 95% CI = [.07, .26], *p* < .001; (ii) stability of self scale, *r* = .14, 95% CI = [.04, .24], *p* = .005; and (iii) self-pluralism scale, *r* = .20, 95% CI = [.10, .29], *p* < .001. Likewise, the adjusted within-person standard deviation in daily personality was positive correlated with (i) the self-concept clarity scale, *r* = .21, 95% CI = [.11, .31], *p* < .001; (ii) stability of self scale, *r* = .18, 95% CI = [.08, .27], *p* < .001; and (iii) self-pluralism scale, *r* = .16, 95% CI = [.06, .25], *p* = .002. Finally, the adjusted within-person standard deviation in self-esteem was positive correlated with the stability of self scale, *r* = .14, 95% CI = [.04, .24], *p* = .007.

As can be seen from comparing the results from Hypotheses 1–3 with the results in the preceding paragraph and the bottom panel of Table 5, very little changes regarding the correlations of the adjusted within-person standard deviation even after the variance associated with response styles is partialled out.

#### Comparison

of the Correlations in Hypothesis 1 With the Correlations Between Average-State and Retrospective Reports

We designed the data collection to answer several unrelated research questions. As such, we have retrospective week-reports of negative and positive affect in the exit survey. This means that, in the same dataset, we can compare the magnitude of the convergent validity correlations in Hypothesis 1 with the magnitude of convergent validity correlations between retrospective reports of affect over the past week and the average of momentary affect reports given throughout that week (i.e., average-state). For the retrospective reports, people were asked to rate how much of each of the emotions in the negative and positive affect scales they experienced during the 1-week experience sampling study (on the same scales, from 1 = not at all, to 5 = extremely). In our data, the average-state correlated quite strongly with the retrospective reports: *r* = .85 for negative affect and *r* = .91 for positive affect. Therefore, there was a correlation of <1, even though one could think of these as measuring the same construct, suggesting that the two measures of affect (average-state vs. retrospective reports) may have different sources of measurement error.

When compared with the convergent validity correlations in Hypothesis 1, the correlations between average-state and retrospective reports were much stronger. The former ranged from .14 to .30, whereas the latter correlations were .85 and .91. This means that even if perceived variability and the adjusted within-person standard deviation are measuring the same construct, their sources of measurement error reduce their convergent validity correlations to a far greater extent than when comparing retrospective reports of affect with the average-state reports of affect. In fact, the convergent validity correlations in Hypothesis 1 are likely to be so low that, as discussed in the discussion section, the results of studies can vary quite substantially depending on which measure is used.

### Summary of Results From the Preregistered Hypothesis Tests

Table 6 summarizes the results of each hypothesis test that was preregistered. The blank cells mean that the relationship was not relevant for the hypothesis, and an “x” indicates that the relationship for that test was not statistically significant.

**Table 6.** Summary of Results From Each Hypothesis Test.

Hypothesis 1		
Emotional Variability	Daily NA SD_adjusted_	Daily PA SD_adjusted_
BFI2 Negative Emotionality	.21	
BFI2 Emotional Volatility	.22	
BFAS Emotional Volatility	.23	
Affect Lability Scale	.30	.30
Hypothesis 2		
Personality Variability	Contextual Personality SD_adjusted_	Daily Personality SD_adjusted_
Self-Concept Clarity	.17	.22
Stability of Self	.15	.18
Self-Pluralism	.20	.15
Hypothesis 3		
Self-Esteem Variability	Daily Self-Esteem SD_adjusted_	
Stability of Self	.14	
Hypothesis 4		
Response Styles	Baseline Image SD_adjusted_	Daily Image SD_adjusted_
Daily NA adjusted *SD*	x	x
Daily PA adjusted *SD*	x	x
Context Pers adjusted *SD*	.13	x
Daily Pers adjusted *SD*	x	x
Daily Self-Esteem adjusted *SD*	x	x

## Discussion

Research has found that different measures of within-person variability lack convergent validity with one another. Self-report measures of perceived variability in a construct tend to correlate only weakly with the within-person standard deviation of repeated reports of the same construct, once the standard deviation is adjusted to account for dependence on the mean (Baird et al., 2006, 2017; Hisler et al., 2020; Kalokerinos et al., 2020; Nestler et al., 2021; Sperry & Kwapil, 2020; Wendt et al., 2020). To explain this lack of convergence, researchers have suggested that repeated reports on the same items, and thus all indices of variability derived from the within-person standard deviation of the repeated reports, are biased by response styles (Baird et al., 2017; Nestler et al., 2021). However, evidence in support of the response styles hypothesis has been inconclusive. In a large experience sampling study, we tested (i) whether there was any convergent validity between perceived variability and an adjusted within-person standard deviation (Hypotheses 1–3) and (ii) the response styles hypothesis (Hypothesis 4). Although the two measures of variability showed some convergent validity, the correlations were rather small and, out of the 10 critical tests of the response styles hypothesis, only 1 was statistically significant at the corrected alpha level. We discuss these findings in turn.

For affect (both positive and negative), personality, and self-esteem, there was some convergent validity between the two measures of variability. The correlations were small, but they were consistently positive and statistically significant. Our findings are thus largely in line with past research on affect showing small or nonsignificant relationships between self-reports of perceived variability (i.e., Neuroticism or Affect Lability) and the within-person standard deviation after accounting for the mean (e.g., Hisler et al., 2020; Kalokerinos et al., 2020; Sperry & Kwapil, 2020; Wendt et al., 2020). Although past research has found nonsignificant relationships between the two measures of variability for personality ratings (Baird et al., 2006, 2017; Nestler et al., 2021), our larger sample size was better suited for reliably detecting smaller effect sizes. Our findings for self-esteem are in line with previous research also finding a small and positive significant correlation (Baird et al., 2017). Taken together, our results suggest that perceived variability in a construct and an adjusted within-person standard deviation in repeated self-reports of the same construct are, at the very least, measuring things that are somewhat related.

However, the correlations between the two measures of variability in affect, personality, and self-esteem were quite small, ranging from *r* = .14 to .30. Such correlations are far too weak for the measures to be used interchangeably: weaker correlations between two measures makes it more likely that there will be a greater difference between the correlations of these two measures with a third measure (Carlson & Herdman, 2012). Indeed, our exploratory analyses showed that self-report measures of well-being were more strongly and consistently correlated with the self-report measures of perceived variability than with the adjusted within-person standard deviation measures of variability, which is in line with past research (e.g., Baird et al., 2006; Fukushima & Hosoe, 2011). But this does not suggest that the perceived variability measures are better, since their correlation with the well-being measures may be due to various forms of common methods bias (Podsakoff et al., 2003). Indeed, there is even content overlap between some of the items in the measures in the baseline survey. For example, some of the items measuring trait negative affect are very similar to the items measuring perceived variability in negative affect (i.e., Neuroticism). Taken together, therefore, studies’ conclusions will vary depending on which measure of variability researchers use.

With this in mind, in our research, we also aimed to investigate a potential source of bias in the measures. Some recent research has suggested that people’s repeated reports, and thus any index of variability based on these, are biased by response styles (Baird et al., 2017; Nestler et al., 2021). Such bias could explain the lack of strong convergence between the adjusted within-person standard deviation and self-reports of perceived variability. However, previous research testing the response styles hypothesis has shortcomings and alternative explanations which we addressed in our study. With our relatively large sample size, we had statistical power to reliably detect much smaller effect sizes than previous studies. We also took ratings for images that should theoretically not be associated with any of the substantive measures for any reason. Therefore, if people’s repeated reports are biased by response styles, even to a relatively small degree, our study would detect positive correlations between the within-person standard deviations in the substantive measures and the image ratings (Hypothesis 4).

Out of the 10 critical tests of Hypothesis 4, only 1 was statistically significant at the corrected alpha level. Therefore, the results from testing Hypothesis 4 provide very little evidence for response styles as a source of systematic measurement error in indices of within-person variability calculated using repeated self-reports. Our results suggest that even if response styles do cause any bias, the bias—at least as measured by the procedure we used—is too small, as shown by the partial correlation analyses, to change anything substantial in terms of how much the index of within-person variability using repeated self-reports correlates with the self-reported variability in the corresponding construct or with any of the outcome variables.

We note that the experience sampling phase of our study lasted for only 1 week. Moreover, we only had 1 or 2 measurement occasions per day for the self-esteem and personality constructs, respectively. One limitation, therefore, is that within-person variability during that 1-week period may not be representative of a person’s variability generally. For example, a person may be more variable in general than they were in that week. And people’s self-reported perceived variability may have been developed over an extended period of time. This means that a lack of convergent validity between the measures could be explained by the short experience sampling phase and too few measurement occasions during each day. On the other hand, our results for negative affect are in line with findings from past research that included a 1-month daily diary study and 3 14-day experience sampling studies with between 5 and 7 measurements per day (Kalokerinos et al., 2020). As such, we think that the relatively low number of measurements are likely to produce similar results to a study with more measurements.

Upon reflecting on our findings, we think it plausible that results from past research taken as support for the response styles hypothesis (Baird et al., 2017; Nestler et al., 2021) could have an alternative explanation. The correlations between the adjusted within-person standard deviation in ratings for many different constructs (e.g., affect, personality, self-esteem, Simpsons characters, satisfaction with neutral objects, and even ratings for the weather) may reflect variability in a broader substantive construct. Namely, an evaluative tendency. Affect and personality items have evaluative content—they consist of items that are either desirable or undesirable. Research has found that this evaluative content may contribute to what has been called a general factor of personality, reflecting intercorrelations between ratings across all items on personality inventories (Wood et al., 2022). Therefore, the correlations between the indices of within-person variability for constructs with items that have evaluative content may reflect within-person variability in an evaluative tendency. Although past research and our results are consistent with such an idea, future research can test this hypothesis more thoroughly.

Given that the different measures of within-person variability show low convergence with one another, what should researchers do when interested in measuring variability? A good starting point is for researchers to consider what kind of variability is best represented by their research question and/or the timeframe of variability that best reflects their population of interest. For example, do the researchers want to measure moment-to-moment fluctuations, day-to-day, week-to-week? Ideally, perhaps, the researchers would then measure the construct repeatedly, based on the timeframe of interest, and use an adjusted within-person standard deviation. For moment-to-moment fluctuations, experience sampling methods with multiple state reports throughout each day should be used; for day-to-day fluctuations, daily diary studies with retrospective reports covering the past day; and for week-to-week, weekly diary studies with retrospective reports covering the past week. The length of such studies would ideally be as long as possible, given resource constraints, so that the time period of the study can be representative of the participants’ lives. However, the least time-intensive method would be to use self-reports of perceived variability. In drawing conclusions from their studies, researchers should take into account that self-report measures of perceived variability are only weakly related to the adjusted within-person standard deviation measures.

## Conclusion

Using a relatively large experience sampling study, we found a consistent but small degree of convergent validity between self-reports of perceived variability and the adjusted within-person standard deviation of repeated self-reports given across contexts or over time. However, for every construct, the correlations between the two measures were too weak for the measures to be used interchangeably. Therefore, researchers will reach different conclusions about the links of variability in affect and personality with well-being, depending on whether they use self-report measures of perceived variability or the adjusted within-person standard deviation of repeated self-reports.

We found very little evidence to suggest that indices of variability calculated using people’s repeated self-reports on the same construct are biased by response styles. Our results suggest that if variable response styles do bias indices of within-person variability, then this is likely to be a trivially small bias that changes almost nothing with respect to how strongly the indices correlate with other measures—at least with respect to bias as measured by the procedure we used. Response styles as a type of systematic measurement error is therefore unlikely to be the main cause of the weak (and sometimes non-existent) convergence between measures of perceived variability and the adjusted within-person standard deviation.

## Notes

1. We also conducted the correlation analyses testing Hypothesis 4 with each image type separately, rather than aggregating the variability in ratings across images. Only the correlation between the adjusted within-person standard deviation in daily positive affect ratings and the adjusted within-person standard deviation in daily color image ratings was statistically significant at the .005 alpha level (*r* = .14, *p* = .0045). Therefore, our results show very little evidence to suggest bias in people’s repeated self-reports is robust.
